# Comparison 30-day clinical complications between transfemoral versus transapical aortic valve replacement for aortic stenosis: a meta-analysis review

**DOI:** 10.1186/1749-8090-8-168

**Published:** 2013-07-03

**Authors:** Xuebiao Li, Minjian Kong, Daming Jiang, Aiqiang Dong

**Affiliations:** 1 Cardiaovascular surgery, Department of second affiliated hospital, school of Medicine, Zhejiang university, No. 88, Jie fang road, Hangzhou, Zhejiang province 310009, China

**Keywords:** Transcatheter aortic valve implantation, Transfemoral, Transapical, Aortic stenosis, Meta-analysis

## Abstract

**Background:**

Since 2002, transapical aortic valve replacement has been developed as a clinical pathway for transcatheter aortic valve implantation (TAVI). However the appropriate role of TA in the AS population versus TF remains unclear. We performed a meta-analysis to assess if TF has any benefit in reduction of 30-day clinical complications in AS.

**Methods:**

We conducted a comprehensive search on pub-med and web of knowledge from 2002 through September 2012 using following terms: aortic stenosis, aortic valve replacement, transcatheter aortic valve implantation, TAVI, trans-artery, transfemoral, trans-apical. Studies in the original research or review articles were also considered. Included studies must meet the preconditioned criterias. Two investigators independently browsed the studies by title and abstract, finally making decision according to full-text. Disagreements were discussed in group.

**Results:**

A total of 20 studies met inclusion criteria’s and were included in the analysis (including 4267 patients in TF group, 2242 in TA group). No random clinical trial, one was a retrospective study, others were prospective trials. Our meta-analysis found that TF had the low incidence of 30-day mortality compared with TA procedure (7.5% versus 11.3%). The incidence of stroke at ≤ 30 days was relatively low (3.8% in TF versus 4.0% in TA). Although the incidence of post-operative heart block was high (8.5% versus 7.5%), but no differences were indicated [1.06,95% CI(0.85,1.33)].

**Conclusions:**

The result of our meta-analysis suggested that TF may have a low risk for 30-day mortality against TA procedure. No difference was found in the incidence of post-operative stroke and heart block.

## Background

Aortic valve disease is the most common acquired valvular disease in developed and developing countries. Surgical aortic valve replacement is the gold standard treatment for patients with aortic stenosis. However, patients in high risk with severe, symptomatic aortic stenosis arenot candidates for surgical AVR. Since encouraging early results from various centers, TAVI is increasingly seen and accepted as an alternative procedure in high-risk patients [1-3].

The TF approach was used in 66% of procedures, but in case of small size and atheromatosis of the iliac arteries, this approach is not suitable. The transapical approach is usually applied for patients with small, calcified,iliac arteries. But the choice of the optimal access route is multifactorial. Patients with inadequate iliac access are usually considered asa high risk associated with bleeding, false aneurysm, or orther vascular complications, which critically influencing the outcome of the patient.

Currently, no randomized studies have been published on the comparison of the TF versus the TA approach. In our work, we aimed to compare 30-day complications in patients with TF against a group of TA.

## Methods

### Search strategy

We conducted a search on pub-med and web of knowledge from 2002 through September 2012 using following terms: aortic stenosis, aortic valve replacement, transcatheter aortic valve implantation, TAVI, trans-artery, transfemoral, trans-apical. Studies in the original research or review articles were also considered.

### Selection criteria

Included studies must meet the following criteria: (a) the studies must clearly describe the study design, country, year of publication, end point. (b) Baseline characteristics of patient in each study must be present (c) Follow-up time is also needed.

### Data extraction

Two investigators independently browsed the studies by title and abstract, finally making decision according to full-text. Disagreements were discussed in a group. We extracted the following information from each study: first author, year of publication, study population characteristics, study size, study design, inclusion and exclusion criteria, time of follow-up, and survival data.

### Data analysis

ORs and their 95% CIs were used to assess the difference between two groups. The heterogeneity assumption was assessed by an I-squared test. If the I^2^ < 50%, then we thought the heterogeneity was not significant, and the fixed effect model was used, otherwise, the random effect model was used.

We first assessed possible sources of heterogeneity within selected studies. For sensitivity analyses, we examined whether excluding studies with substantial deviation from Hardy –Weinberg equilibrium affected our pooled estimates to find if our result is stable. Finally, we assessed publication bias using funnel plots.

We used Cochrane Collaboration meta-analysis software, Review Manager4.2 to perform data analysis. And data was presented as mean ± SD, P value of 0.05 for any test or model was considered to be statistically significant.

## Results

### Characteristics of selected studies

**Figure 1:**
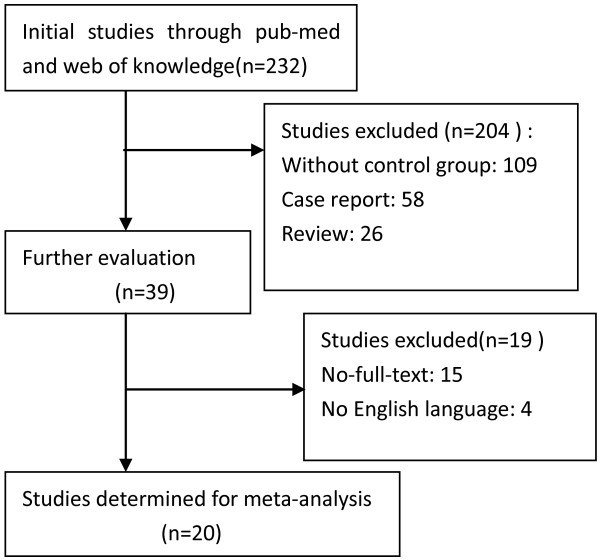
Process for selecting final studies.

A total of 20 studies met inclusion criterias and were included in ouranalysis (4267 patients in TF group, 2242 in TA group) [1-20]. No random clinical trial, one was a retrospective study, others were prospective trials. Table 1 showed the characteristics of included studies. Figure 1 showed the process for selecting final studies.

**Table 1:** Characteristics of included studies

**Study**	**Number of patients TAVI**			**Logistic EuroSCORE**		**STS SCORE**		**30-day outccomes**							
				(%)*		(%)*		**Mortality**		**Stroke**		**Vascular complication**		**Heart block**	
	**TF**	**TA**	**Total**	**TF**	**TA**	**TF**	**TA**	**TF**	**TA**	**TF**	**TA**	**TF**	**TA**	**TF**	**TA**
Rafal Dworakowski [1]	67	84	151	19.4 ± 1.1	23.4 ± 1.5	-	-	4	11	5	4	11	2	3	5
Peter Wenaweser [2]	27	43	70	22.8 ± 13.0	26.1 ±14.3	6.3 ± 4.0	6.3 ± 5.5	3	4	0	1	-	-	3	7
Cosmo Godino [3]	107	15	222	26.6 ± 16	32.2 ± 23	7 ± 4.9	8.3 ± 4.2	1	2	-	-	-	-	-	-
He’ le ‘ ne Eltchaninoff [4]	161	71	232	-	-	-	-	18	12	7	2	-		22	4
Johan M. Bosmans [5]	99	88	187	29 ± 15	33 ± 17	-	-	5	2	2	7	-	--	4	5
Matthias Thielmann [6]	15	24	39	12.7 ± 0.9	16.7 ± 4.4	15.1 ± 4.1	9.9 ± 7.5	2	5			-		-	-
Nawwar Al-Attar [7]	35	15	50	-	-	-	-	3	4			-	-	-	-
Mark D. Osten [8]	16	30	46	-		-	-	1	2	1	2	-	-	1	3
Ronen Gurvitch [9]	169	101	270	-	-	-	-	13	13	7	2	14	4	10	6
ZHAO Quan-ming [10]	28	20	48	20.6 ± 15.8	17.3 ± 16.1	-	-	1	4	-	-	-	-		
Miriam Puls [11]	83	97	180	-	-	-	-	4	2		-	-	-	37	18
KONSTANTINOS SPARGIAS [12]	59	32	91	26 ± 12	27 ± 15		-	1	2	5	4	-	-	-	-
GERHARD SCHYMIK [13]	174	126	300	21.9 15.9	27.0 18.0	-	-	13	5	-	-	-	-	-	-
Martine Gilard [14]	2361	567	2928	21.2 ± 14.7	24.8 ± 17	14.5 ± 11.9	15.1 ±38.8	190	77	-	-	-	-	-	-
Himbert [15]	51	24	75	25 ±13	28 ± 13	15 ± 7	18 ± 9	4	4	3	10	6	2	3	1
Rodés-Cabau [16]	168	177	345	-	-	9.0 ±5.8	10.5 ± 6.9	16	20	5	3	-	-	6	11
Malin Johansson [17]	10	30	40	25.6 ±15	23.5 ± 17	-	-	2	2	2	1	-	-	0	0
Martyn Thomasp [18]	463	575	1038	25.7 ± 14.5	29.1 ± 16.3	-	-	29	59	11	16	106	27	31	42
ThierryLefe’ vre [19]	61	69	130	25.7 ± 11.5	33.8 ±14.4	11.3 ± 6.1	11.8 ± 6.8	5	3	2	1	17	3	1	2
John G. Webb [20]	113	55	168	-	-	-	-	9	10	6	1	9	2	5	4

*TAVI*, transcatheter aortic valve implantation; *TF*, transfemoral; *TA*, transapical; *STS*, Society of Thoracic Surgeons.

*Mean±SD.

Odds for 30-day clinical outcomes compared transfemoral approach with transapical approach in aortic valve replacement.

#### Postoperative 30-day mortality

**Figure 2:**
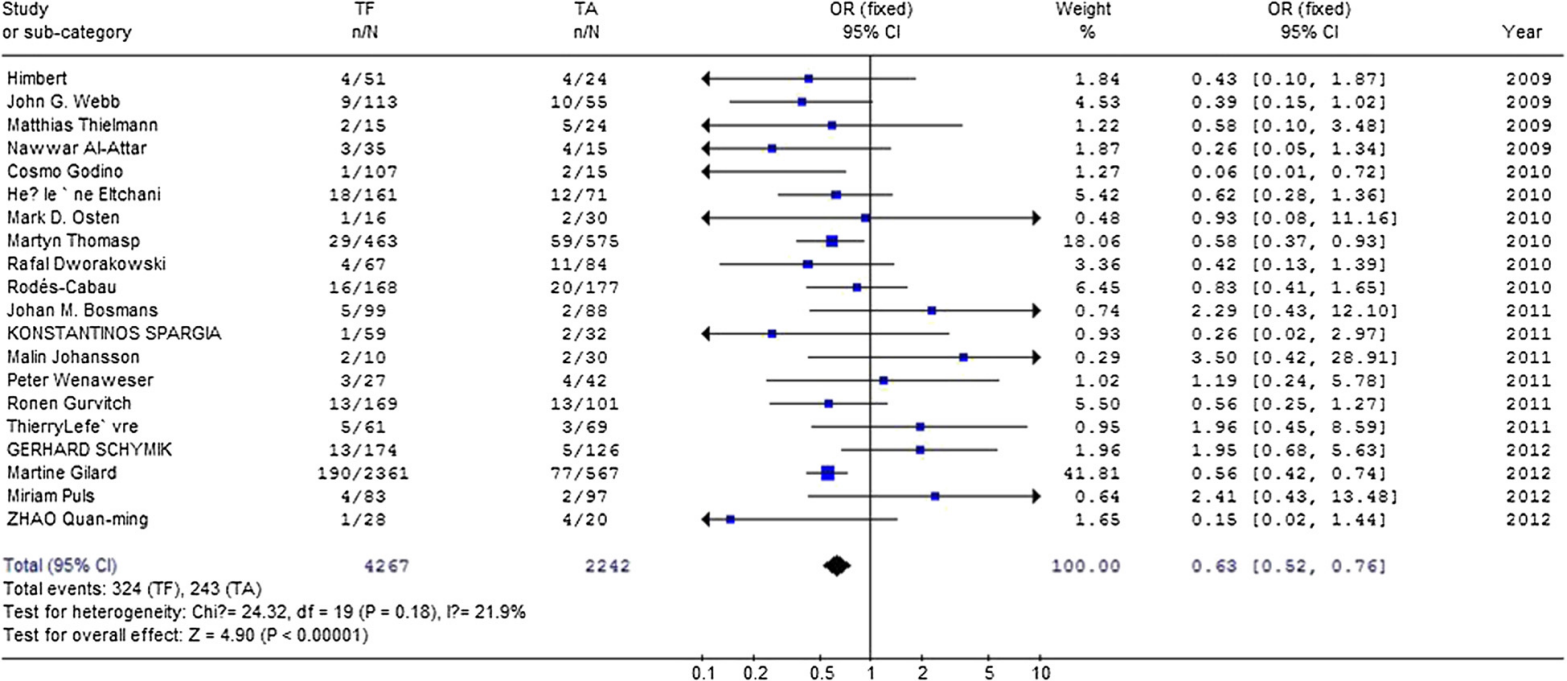
Compare 30-day mortality between transfemoral aortic valve implantation versus transapical surgery aortic valve replacement for aortic stenosis.

**Figure 3:**
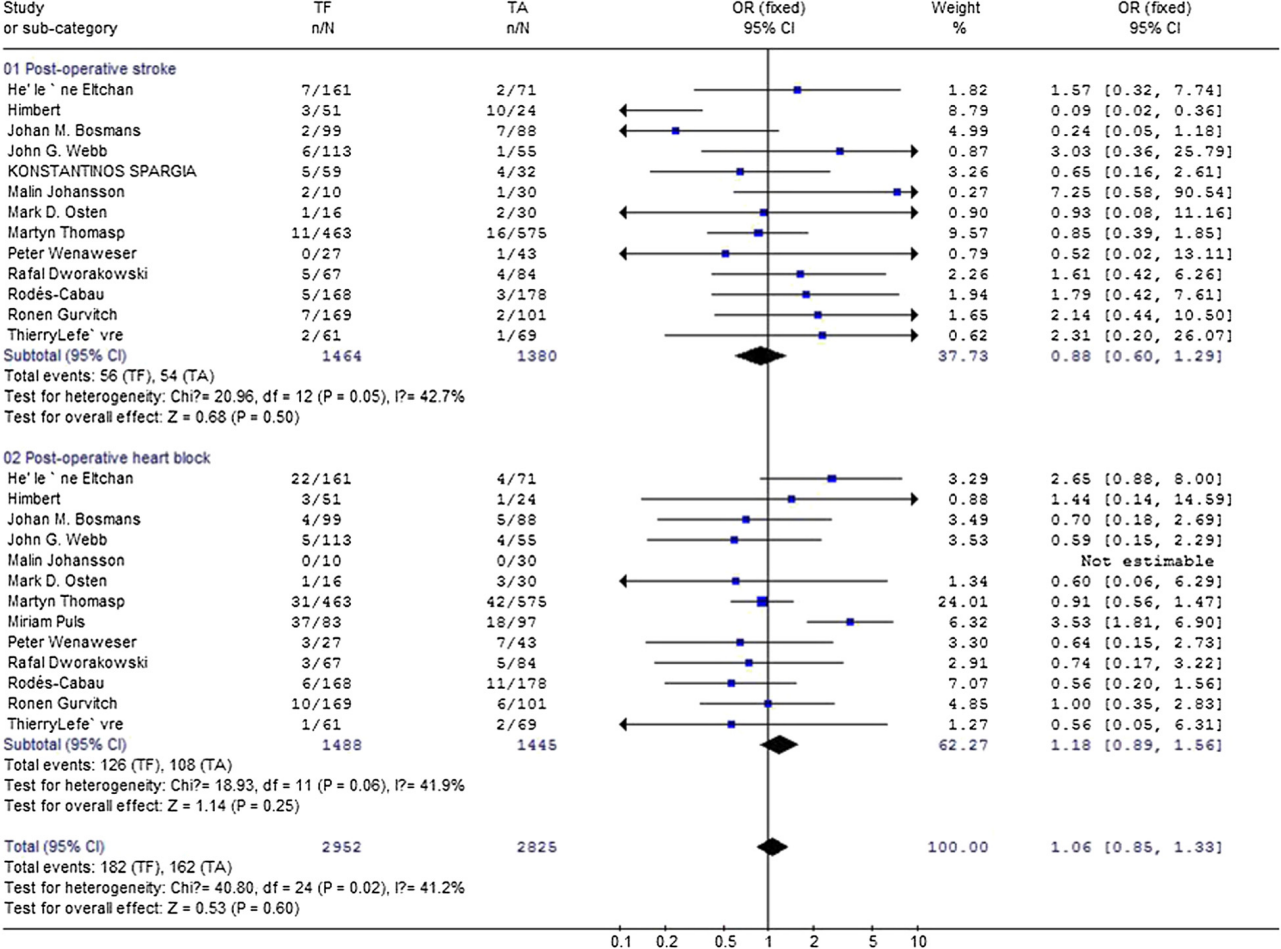
Compare 30-day post-opertive stroke,heart block between transfemoral aortic valve implantation versus transapical surgery aortic valve replacement for aortic stenosis.

The postoperative mortality was reported in 20 studies [1-20] and meta-analysis of the data showed the pooled postoperative mortality in TF was 7.5% (315/4267) as compared with 11.3% (253/2242) in TA. As shown in Figure 2, differences of postoperative mortality reach statistical significance [0.63, 95%CI (0.52, 0.76)]. SAPIEN bovine valve was used when patient receive TA and TF procedure. In small studies, Core Valve were used when patient receive TF procedure. As shown in Figure 3, we performed meta-analysis with studies in which only SAPIEN bovine valve was used and found that postoperative mortality was 7.3% (116/1590) as compared with 11.0% (157/1421) in TA.

#### Post-operative stroke

**Figure 4:**
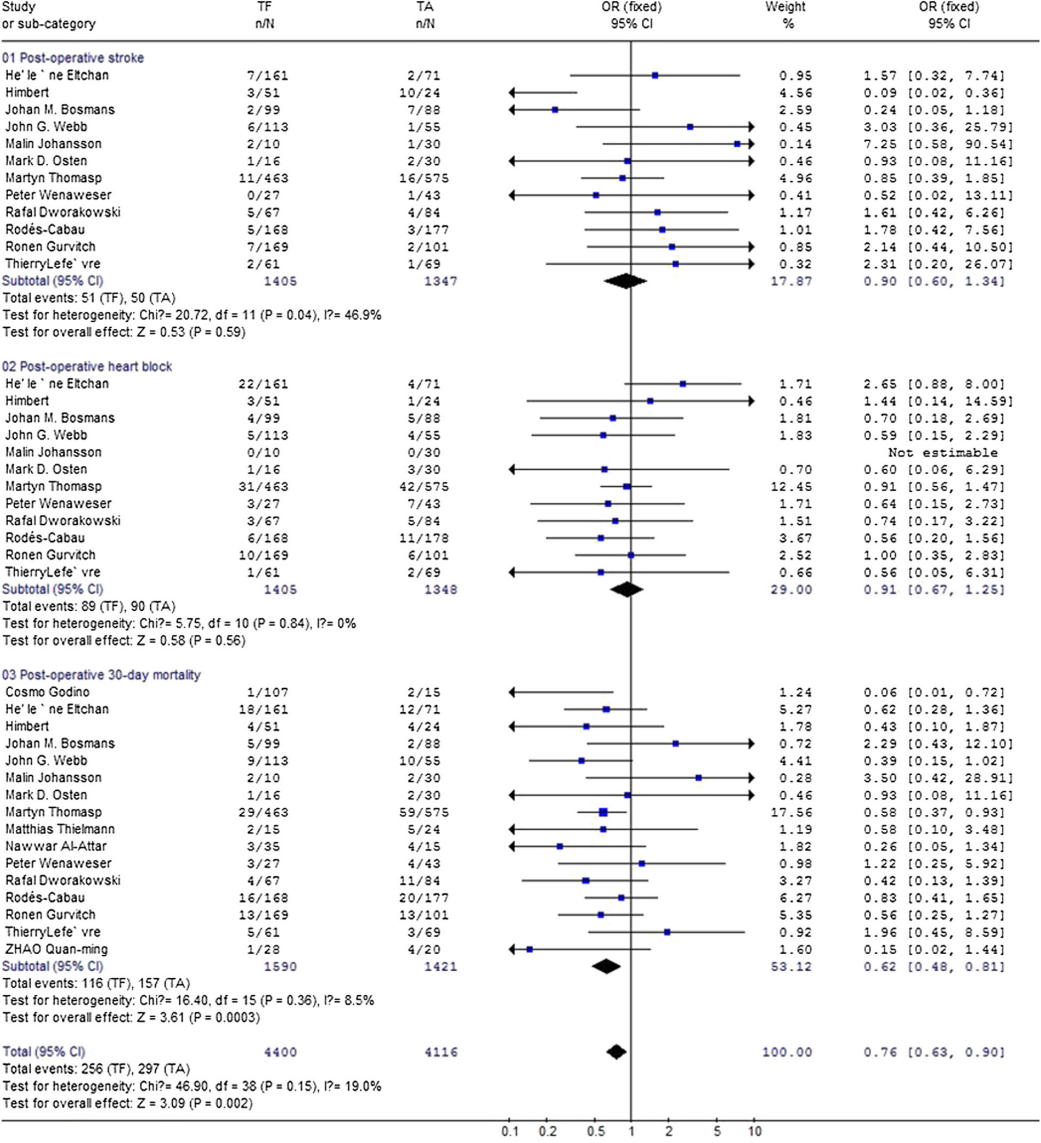
Compare 30-day post-opertive mortality, stroke, heart block between transfemoral aortic valve implantation versus transapical surgery aortic valve replacement for aortic stenosis with studies in which people received only SAPIEN bovine valve was used.

Thirteen studies [1,2,4,5,8,9,12,15-20] reported the postoperative stroke in patients ,meta-analysis of the resultant data showed the pooled postoperative mortality in TF was 3.8% (56/1464) as compared with 4.0% (54/1380) in TA, but did not reach statistical significance [0.88,95%CI(0.60,1.29)], as shown in Figure 3. This finding was consistent with previous study, where the overall 30-day stroke was 3.3 ± 1.8%. There is evidence showed that valve type may influence the incidence of postoperative stroke [21]. We conducted a meta-analysis with studies where only SAPIEN bovine valve was used ,and found postoperative stroke in TF was 3.6% (51/1405) compared with 3.7% (50/1348) in TA., but did not reach statistical significance [0.90,95% CI(0.60,1.34)](as shown in Figure 4).

#### Post-operative heart block

Differences of postoperative heart block were extracted from 13 studies [1,2,4-5,8-9,11,15-20] and meta-analysis of these data showed the pooled postoperative heart block in TF was 8.5% (126/1488) as compared with 7.5% (108/1445) in TA, but did not reach statistical significance [1.18,95% CI(0.89,1.56)], as shown in Figure 3. There is evidence that patients who undergo catheter valve implantation with the CoreValve prosthesis are at higher risk for post-operative heart block [5]. In 7 studies, both Edwards SAPIEN bovine valve and Core Valve were used when patient receive TF procedure. Then we conducted meta-analysis with studies in which only SAPIEN bovine valve was used. Data were extracted from 12 studies and meta-analysis of these data showed the incidence of postoperative heart block in TF was 6.3% (89/1405) compared with 7.5% (90/1348) in TA., but did not reach statistical significance [0.91,95% CI(0.67,1.26)](as shown in Figure 4).

### Sensitivity analysis

Among the studies included in our meta-analysis, no random clinical trials, one was a retrospective study and others were prospective study. We first excluded the retrospective study to find if study-design may influence the result, then excluded studies with large ORs. Except the reducing of I^2^ value, most pooled estimates were similar and did not change materially. There is evidence show that valve type may influence the incidence of postoperative stroke and heart block [21]. We conducted a meta-analysis with studies where only SAPIEN bovine valve was used, and indicated that our results were consistent.

### Publication bias analysis

**Figure 5:**
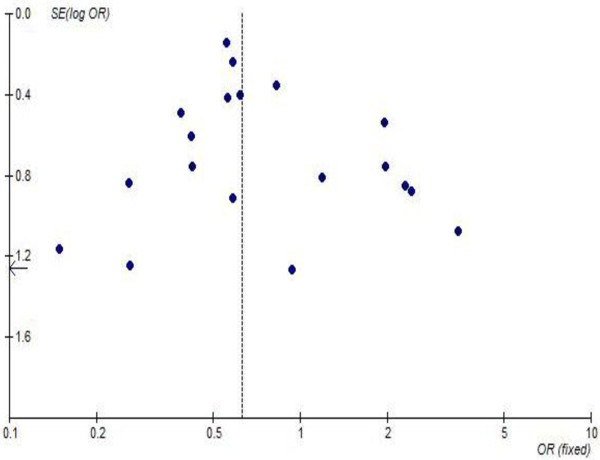
Funnel plot test of the 11 studies included in our meta-analysis.

Funnel plot was used to assess the publication bias. Figure 5 showed the funnel plot for all-cause 30-day mortality. Although small studies with large standard errors tended to scatter above the horizontal line for post-operative 30-day mortality, but no publication bias were indicated (P = 0.230 for the Begg test; P = 0.171 for the Egger test). And we found similar pooled estimate for post-operative stroke and heart block with no publication bias (P > 0.2 for the Begg test; P > 0.1 for the Egger test) even after excluding the small studies in which all ORs exceeded 3.0 and had wider CIs [1,2,5,15,16,18].

## Discussion

The Edwards-Sapien bioprosthesis has now been approved for clinical use in the European Union and preliminary guidance for its use has been published by the National Institute of Clinical Excellence. But the CoreValve has the advantage of a smaller introducer size (18 Fr) for TF access in comparison with the 22/24 French size necessary for Edwards SAPIEN valve implantation. This size allows for a larger use of TF implantation in smaller arteries (6.0 mm). The TF approach was used in 66% of procedures [4], the TA approach is an alternative to TF for the Edwards SAPIEN device. Valve implantation was performed using the first generation Edwards SAPIEN prosthesis (Edwards Lifescience, Irvine,CA) or the Medtronics COREVALVE (Med tronics Inc., Minneapolis, MN) in selected studies. Transfemoral use of the Edwards SAPIEN prosthesis was the initial option considered for all patients. In cases of inadequate arterial anatomy (including small vessel diameter, severe calcifications, and/or severe arterial tortuosity), transapical use of the Edwards SAPIEN prosthesis was considered. The Medtronics COREVALVE was preferred for patients with a large aortic annulus (diameter ranging from 23 to 27 mm), in which case the first generation of Edwards SAPIEN prosthesis was contraindicated.

With 4267 patients in TF group, 2242 in TA group from 20 studies, our meta-analysis identified transfemoral patients had a higher 30-day mortality of 7.5% compared with 11.3% in the transapical patients. The reason for this difference is unclear, but we believe that the difference in mortality between two approaches is partially explained by the different risk profiles of the patients and appropriate clinical judgment strategies. Logistic EuroSCORE can be helpful in identifying high risk patients. But it may not be appropriate to extrapolate EuroSCORE algorithms based on operated patients to this TAVI population, the majority of which was deemed inoperable, and other clinical and morphological variables (such as porcelain aorta, frailty, planned multivalve surgery) are not captured by this score.

The incidence of stroke at ≤ 30 days was relatively low, 3.8% in the TF group and 4.0% in the TA group. This may be related with careful patient selection, device preparation, optimal device progression, and positioning, as well as adequate antiplatelet and anticoagulation regimen. It has previously been suggested that the stroke rate for the transfemoral group may be higher than for the transapical group because of the passage of the 22F or 24F sheath around the aortic arch [20]. But we found no significant difference [0.87, 95%CI (0.59, 1.29)].

Incidence of the post-operative heart block comprising sudden post-operative AV block or other cardiac arrhythmia which need pace-maker implantation, is a life-threatening complication. It is speculated that conduction tissue injury during TAVI is induced by mechanical pressure to the conduction system by the prosthesis and the native valve calcium that remains in situ. The risk of post-operative heart block in patients with the Edwards SAPIEN valve prosthesis is lower than that observed in patients with the CoreValve valve prosthesis (6.3% for the TF and 7.5% TA approach).The reasons are unclear but may relate to the fact that the Edwards SAPIEN valve is shorter than the CoreValve device and that there is continued pressure on the conduction system in the septum by the self-expanding CoreValve.

Our study existed several limitations. First, publication bias might have occurred because our study were wholly based on studies published in English-language journals, studies in other language and unpublished studies were not included. Although the pooled estimate did not change after we excluded the smallest studies with large ORs, we cannot completely rule out the possibility that TF advantages were overestimated because of publication bias and patient selection criteria.

Some may also argue that the true effect of two procedures on high risk patient can be quantified from meta-analysis of studies with heterogeneous samples. We dealt this concern primarily by using multiple sensitivity analysis, all of the analysis produced consistent pooled estimates, although false-positive finding are possible in analysis because of small subgroups.

## Conclusions

Our meta-analysis found that TF had the low incidence of 30-day mortality compared with TA procedure (7.5% versus 11.3%). The incidence of stroke at ≤ 30 days was relatively low. Although the incidence of post-operative heart block was high, but no differences were indicated.

## Abbreviations

TF: Transfemoral; TA: Transapical; AVR: Aortic valve replacement; SAVR: Surgical aortic valve replacement; TAVI: Transcatheter aortic valve implantation.

## Competing interests

The authors declare that they have no competing interests.

## Authors’ contributions

X-L carried out the comprehensive search on library database, participated in selecting studies for meta-analysis, and drafted the manuscript. M-K carried out the comprehensive search on library database, participated in selecting studies for meta-analysis and drafted the manuscript. D-J performed the statistical analysis. A-D conceived of the study, and participated in its coordination and helped to draft the manuscript. All authors read and approved the final manuscript.
